# Identification of leader and self-organizing communities in complex networks

**DOI:** 10.1038/s41598-017-00718-3

**Published:** 2017-04-06

**Authors:** Jingcheng Fu, Weixiong Zhang, Jianliang Wu

**Affiliations:** 1 School of Mathematics, Shandong University, Jinan, 250100 China; 2 College of Math and Computer Science, Institute for Systems Biology, Jianghan University, Wuhan, 430056 China; 3 Department of Computer Science and Engineering, Washington University, St. Louis, MO 63130 USA

## Abstract

Community or module structure is a natural property of complex networks. Leader communities and self-organizing communities have been introduced recently to characterize networks and understand how communities arise in complex networks. However, identification of leader and self-organizing communities is technically challenging since no adequate quantification has been developed to properly separate the two types of communities. We introduced a new measure, called ratio of node degree variances, to distinguish leader communities from self-organizing communities, and developed a statistical model to quantitatively characterize the two types of communities. We experimentally studied the power and robustness of the new method on several real-world networks in combination of some of the existing community identification methods. Our results revealed that social networks and citation networks contain more leader communities whereas technological networks such as power grid network have more self-organizing communities. Moreover, our results also indicated that self-organizing communities tend to be smaller than leader communities. The results shed new lights on community formation and module structures in complex systems.

## Introduction

Real systems in various fields, e.g., power grids in engineering, gene regulatory networks in biology, and circles of friends in social media like Facebook, are best formulated as graphs or networks where nodes represent entities and links represent relationships between entities. Complex networks are not random^1, 2^, but rather have various structural and locational properties. One important property common to many networks is the presence of community structures, where the nodes within a community are more densely connected than the nodes across two communities^3^. Communities are an important network property^3–6^ since they typically constitute functional units of a network. For instance, groups in social networks often possess their own norms, orientations and subcultures^7^. Within a cell, some proteins interact to form protein complexes to exert their functions^8, 9^.

Identification of communities in complex networks has attracted much attention^10–12^. Recent studies have extended to discovering internal, fine structures of network communities. Two types of internal communities have recently been proposed, the leader communities and self-organizing communities^13, 14^. Identification of such fine community structures can help gain insights into the formation of communities and provide some deep knowledge of network properties.

A leader community is a community where a few nodes are highly connected to nearly all or a substantial number of other nodes in the community - these highly connected nodes can be viewed as the leaders of the community and have a great influence on the community as a whole. One well-known example of leader communities is Zachary’s karate club network^15^, where the coach and the president of the club went into a dispute, causing the club to split into two with each of them being the leader of a group. In contrast, a self-organizing community is a community where all nodes have nearly equal or similar node degrees - there exists no single node that has dominating influence on the community. One example is the American College Football network where each team within a conference plays against every other team, thus forming a self-organizing community.

**Figure 1:**
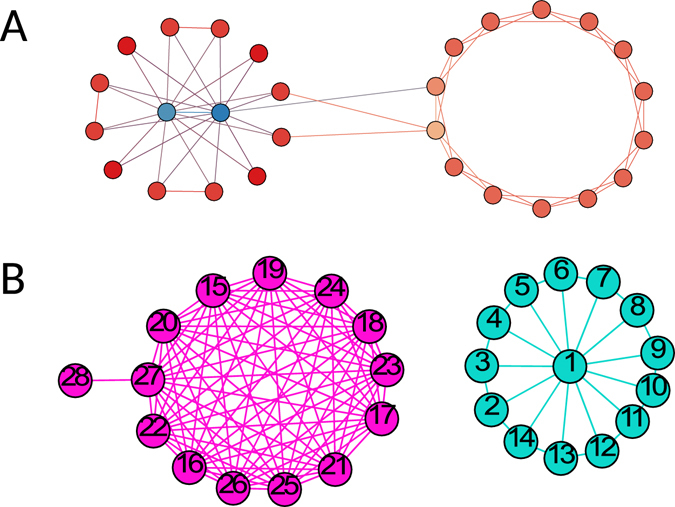
A network with both kinds of communities. (**A**) A leader community (left) and a self-organizing community that can be identified by the variance of node degree. The two communities have 14 nodes and an average node degree of 4. However, the degree variance of the leader community is 13.7 while the degree variance of the self-organizing community is 0. Here the degree of a node counters the edges within its community. (**B**) Another leader community (right) and a self-organizing community (left), each of which has 14 nodes. The leader community has an average node degree of 3.7 and a degree variance of 7.1. In contrast, the self-organizing community has a larger average node degree of 11.3 and a larger degree variance of 8.8. While the self-organizing community has a larger degree variance, it is not a leader community. Likewise, the leader community has a smaller degree variance than the self-organizing community, the former is a typical leader community.

Automatic distinguishing a leader community from a self-organizing community seems to be challenging. One plausible characteristic metric is the variance of node degree^13^. The leaders in a leader community have significantly higher node degrees than the other nodes, whereas the nodes in a self-organizing community have similar node degrees. Therefore, a leader community might have a higher variance of node degree than a self-organizing community. It has been suggested to use *VAR*(*C*)>1 as a criterion to call a community *C* a leader community if its degree variance *VAR*(*C*) is greater than one^13^. One illustrating example is in Fig. 1A, where the left community is a leader community with two leaders in blue and the right community is a self-organizing community without a leader.

While seemed to be reasonable, this variance-based metric is not effective for separating leader and self-organizing communities. One example on which this metric fails completely is in Fig. 1B. As shown, the community on the left is a self-organizing community and the one on the right is a leader community with one leader node. The two communities all have large variances of node degree. The self-organizing community has a variance of 8.8 and the leader community 7.1. Remarkably, the self-organizing community in fact has a larger variance than the leader community! This simple example in part motivated our present study.

We developed a novel metric for automatic identifying and distinguishing leader and self-organizing communities. We studied the utility and robustness of this new metric on various real complex networks, demonstrating its effectiveness in comparison with the variance based metric.

## Results

### The main idea and the new metric

The result in Fig. 1B is thought provoking. Having a large variance of node degree may be a necessary feature, but not a sufficient condition, for forming a leader community. This means that while a leader community typically has a large variance of node degree, a community with a large degree variance may not be necessarily a leader community. This also entails that there must be some hidden feature that separates leader and self-organizing communities apart.

The variance-based metric fails in part because it uses no reference to network structures to adequately call for a leader or a self-organizing community for a given network. It ignores the inherent, albeit hidden, structures embedded in a given community. Instead, it rigidly relies on a fixed criterion of *VAR*(*C*)>1 to call for a leader community and *VAR*(*C*)<1 for a self-organizing community. Here, the threshold “1” used is very much arbitrary.

A leader community has an eminent feature of having a few leaders that are highly connected. Thus, a leader community is sharply different from a random community even if the latter has the same average node degree. A random community resembles a self-organizing community as both of them do not seem to have any particular structures. This observation suggests that a remedy to the serious drawback of using the degree variance alone is to introduce an expected structure that is anticipated for a given community as a reference. To this end, we introduce and utilize a random null model of the community that has the same average node degree as the given community. The variance of the node degree of the random null model is then used as a reference to the variance of the node degree of the given community.

Specifically, we took the ratio *ρ* between the degree variance of the given community and the expected degree variance of the corresponding random model as a yardstick to quantify a community. That is

1 $$\rho =VA{R}_{real}/VA{R}_{rand}$$

where *VAR*_*real*_ and *VAR*_*rand*_ are the degree variances of the actual and random communities, respectively. When *ρ* > 1, the community has a degree variance greater than the expected degree variance of the random model that has the same average connectivity. A larger *ρ* > 1 means that the given community deviates more significantly from the random model, indicating more strongly the former to be a leader community. In contrary, when *ρ* < 1, the given community has a more uniform node degree than the random model, so that the former is more likely to be a self-organizing community. When *ρ* = 1, the community can be viewed as neither a leader community nor a self-organizing community.

### The Erdös-Rényi(ER) random network and variance of degrees

**Figure 2:**
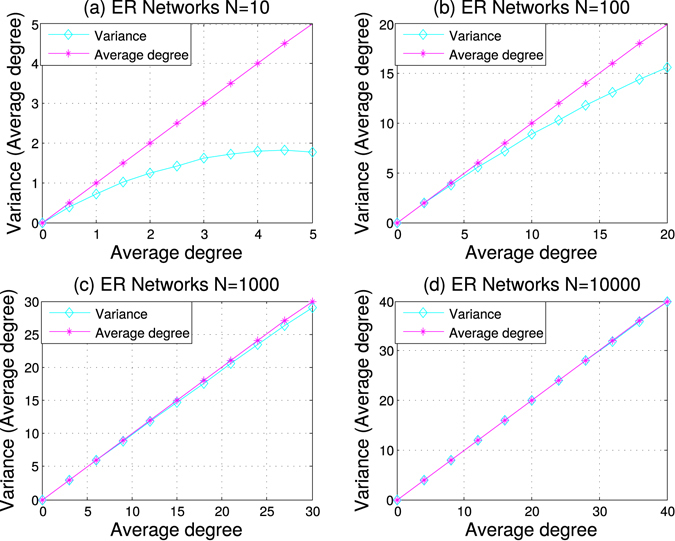
Approximation of the expected degree variance with the average node degree in the ER network. This experimental analysis of the relationship between the expected variance of node degrees and the average degree was done on networks of four increasing sizes, *N* = 10, 100, 1000 and 10,000, with the average degrees of [0.5, 1, 1.5, …, 5, [2, 4, 6, …, 20], [3, 6, 9, …, 30], and [4, 8, 12, … 40], respectively. Each data point in the figure was averaged over 1,000 random problem instances.

The new method uses a random null model as a reference. To this end, we adopted the Erdös-Rényi(ER) random network^16^ as the random model. An ER random network with *N* nodes and an average node degree 〈*k*〉 can be generated by connecting a pair of nodes with probability of 〈*k*〉/(*N* − 1). One useful feature of the ER network is that its expected variance of node degrees can be efficiently and accurately approximated using *N* and 〈*k*〉 (see Methods). It can also be shown that the expected variance of node degrees is not greater than the average node degree (see Methods). Furthermore, these two values become closer to each other as the average degree decreases and the network becomes larger. In other words, the sparser the network, the closer the two values (Fig. 2).

### Evaluation of the new metric

In order to assess the new metric *ρ* for separating leader and self-organizing communities, we applied it to several well-known real networks with known community structures. These real networks include the Dolphins network^17^, Football network^18^, Karate network^15^, Polblogs network^19^, Polbooks network^20^, School friendship network^21^ and Word network^22^ (Table 1 and Materials and Methods).

**Table 1:** Eight real networks with known community structures.

Name	n	m	K	${N}_{leader}^{old\,method}/{N}_{self\,organizing}^{old\,method}$	${N}_{leader}^{new\,method}/{N}_{self\,organizing}^{new\,method}$	Ref
Dolphins	62	159	2	2/0	2/0	17
Karate	34	78	2	2/0	2/0	15
Polblogs	1,222	16,714	2	2/0	2/0	19
Polbooks	105	441	3	2/1	2/1	20
Word	112	425	2	1/1	1/1	22
Football	115	613	12	2/10	3/9	18
School6	69	440	6	5/1	6/0	21
School7	69	440	7	5/2	7/0	21

In the table, *n* and *m* are the numbers of nodes and edges, respectively, and *K* is the number of communities in a network. ${N}_{x}^{y}$ means the number of x community under method y, where x ∈ {leader, self organizing} and y ∈ {old method, new method}.

**Figure 3:**
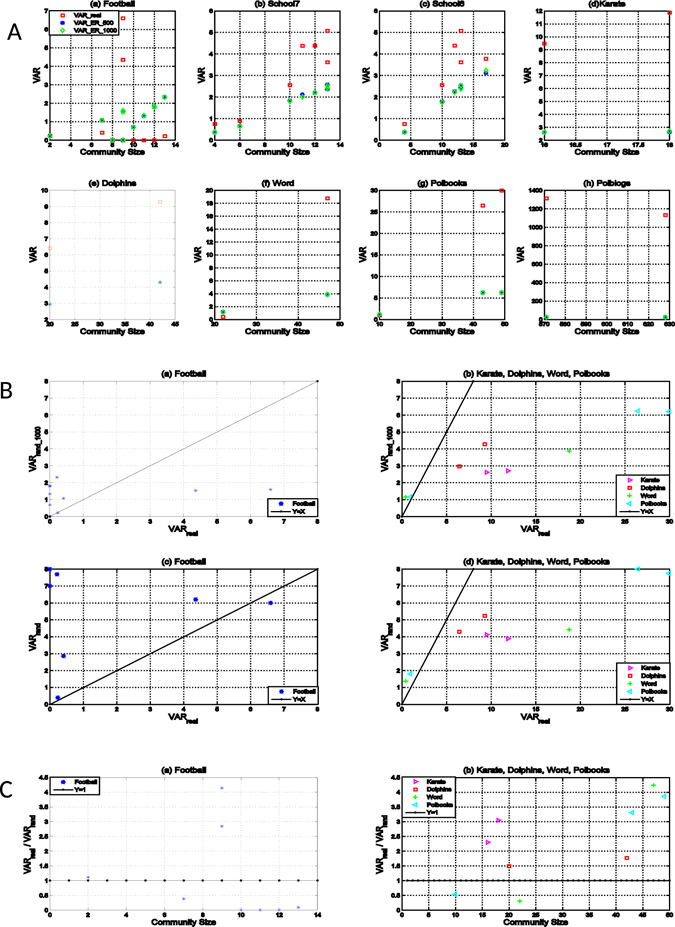
*VAR*_*real*_’s, *VAR*_*rand*_’s and variance ratios of communities in real world networks with known community structures. Here *VAR_real* is the *VAR* of a community. *VAR*_*rand*_’s in panel A are from simulation, averaged over *M* randomly generated ER networks with the same number of nodes *N* and average node degree, where *M* takes values of 500 and 1,000 and denoted as *VAR_ER_*500 and *VAR_ER_*1,000, respectively. As shown, *VAR_ER_*500 is almost the same as *VAR_ER_*1,000, suggesting that the approximation of *VAR*_*rand*_ via simulation is stable and efficient. Panel B shows the results from simulation and approximation *VAR*_*rand*_ = 〈*k*〉. Simulation with *M* = 1,000 is used for *B*(*a*) and *B*(*b*) and approximation for *B*(*c*) and *B*(*d*). The line in each diagram is the bound of *VAR*_*rand*_ = *VAR*_*real*_. The ratios of *p* = *VAR*_*real*_/*VAR*_*rand*_ are shown in panel C, where *VAR*_*rand*_’s are from simulation with *M* = 1,000 as in B. The x-axes of the figures correspond to community sizies, which are ordered from small to large.

Each of the Dolphins network, Karate network, Polblogs network and Word network has two communities, and the Polbooks network has three. Two of the communities of these five networks, the first community of the Polbooks network and the third community of the Word network, have real values of degree variance smaller than $1$, indicating that these two communities are self-organizing communities under the old metric (Fig. 3A). On the other hand, the real degree variances of these two communities are respectively smaller than the expected degree variances of their corresponding random graphs. They are detected as self-organizing communities under the new metric as well (Fig. 3B(b)). These two communities are sparse and all of their nodes have similar degrees, which make the real variances not only smaller than $1$ but also smaller than random variances. The real variances are greater than the random variances for the rest $9$ communities of these five networks, meaning that they are classified as leader communities under the new metric. On the other hand, all these $9$ communities have real variances greater than $1$ and should be leader communities under the old metric. Because each of these $9$ communities has at least one leader node with a high degree, which makes its real variance large enough (greater than $1$ and the random variance). In short, the old and new metrics had the same results on these networks regarding the types of communities they have (Table 1).

The disagreement between the two metrics appeared in the last three networks. The Football network is special. Three known communities in this network, corresponding to the conferences of “Conference USA”, “Independents” and “Western Athletic”, do not have well organized structures. Each of these communities has at least one team (node) played more games with teams in the other conferences than in its own conference. The new metric was able to detect this anomaly and correctly mark these communities differently from other communities (Table 1 and Fig. 3C(a)). However, the old metric could only detect some of these anomalous communities. It correctly detected two leader communities, the conferences of “Conference USA” and “Independents”, but failed to identify the conference of “Western Athletic” as a leader community.

The School6 and School7 networks are the same but are divided into 6 and 7 communities, respectively. For these two networks, $VA{R}_{real}$’s are greater than $VA{R}_{rand}$’s for all communities (Fig. 3A(c) and A(b)), meaning that all communities in these networks were marked as leader communities using the new metric. However, two communities in the School7 network were recognized as self-organizing communities by the old metric. These two communities, on which the two metrics gave different results, are shown in Supplemental Figure S1. One of these two communities forms a simple star with total four nodes and the node with degree 3 being the leader. The other community has a leader as well, which is the node with degree 5. The old metric made the same mistake on the School6 network to mark a leader community as a self-organizing community (Figure S1(a)).

In summary, the new metric evidently outperformed the old metric on these real networks.

On large networks, it is computationally inefficient to derive $VA{R}_{rand}$ via simulation (Fig. 3B). We thus have to adopt the approximation scheme $VA{R}_{rand}\approx \langle k\rangle$ (see Methods). We chose five networks to show the effects of using $VA{R}_{rand}$’s from simulation and from approximation $\langle k\rangle$ (Fig. 3B). All communities were classified correctly by the values from the two methods, except two special cases. The Football network was reported to have three leader communities when $VA{R}_{rand}$’s were derived from simulation (3B(a)), whereas only one of these communities was detected as a leader community by the approximation method $\langle k\rangle$ (3B(c)). One of the two communities with disparity results is dense so that the approximation $\langle k\rangle$ is not accurate. The other case is a disconnected community that has five nodes and one edge, on which a corresponding random ER network is more likely to have no edge and the $VA{R}_{rand}$ from simulation may be smaller than its real variance. Despite these three exceptional communities with ill-organized structures, the approximation of $\langle k\rangle$ performed well on most large real networks.

In summary, the results on these real networks with known community structures evidently showed that the new metric is able to distinguish subtle community features that would have otherwise been missed by the old metric, demonstrating the superiority of the new metric over the old one.

### Applications of the new metric

To further analyze the new metric, we applied it to real-world networks with little information of community structures, aiming at revealing deep structural properties of these real networks.

The analysis was carried out in comparison with $9$ existing community detecting methods on $4$ large real-world networks with no information of community structures. These methods include BGLL^23^, RB^24^, CPM^25^, SCluster^26^, UVCluster^27^, OSLOM^28^, SVI^29^, Infomap^30^ and COPRA^31^. BGLL is regarded as one of the best algorithms for community detection based on modularity maximization, and can detect hierarchical community structures. Both RB and CPM are based on a multi-resolution Potts model. Consensus hierarchical clustering is used in both SCluster and UVCluster, along with two methods to maximize a function called Surprise^32^. OSLOM is an algorithm based on optimization of a local fitness function. SVI is based on a Bayesian network model that allows nodes to be members of multiple communities. Therefor, it can detect overlapping community. Infomap, which is regarded as the fast method to detect communities in direct networks, is based on information theory. The last method, COPRA, is based on the well-established label propagation. It can also detect overlapping communities.

The real-world networks that we considered are US airport network, Power grid, Ca_Hepth citation network and Pretty-Good-Privacy social network (PGP network) (Table 2). Pretty-Good-Privacy is an encryption algorithm for safe communication, and the PGP network is a social network in which a node represents a user on the web and an edge between two users means that they trust each other. This network was built based on the data of $\mathrm{191,548}$ keys and $\mathrm{286,290}$ signatures which are generated using the PGP algorithm.

**Table 2:** Information of Real-world Networks with no known community structures.

Name	n	m	Description	Ref
US airport network	500	2,980	Transportation network	33
Power grid network	4,941	6,594	Technology network	34
Ca_Hepth	9,877	51,971	Citation network	35
PGP network	10,680	24,340	Social network	36

Here, *n* and *m* are, respectively, the numbers of nodes and edges in network.

**Figure 4:**
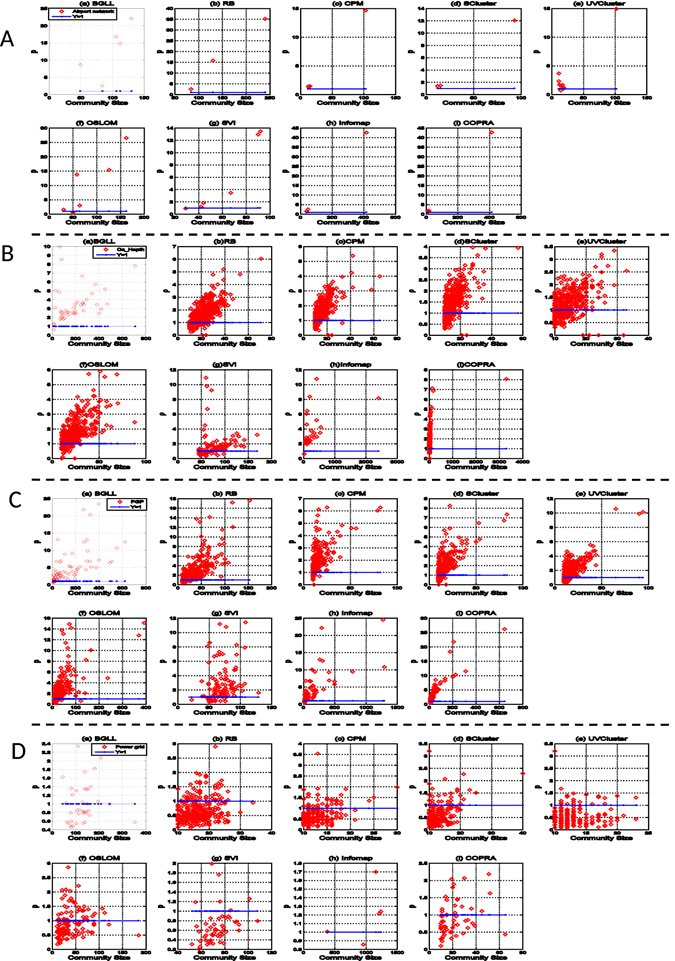
Variance ratios *ρ* of communities in (**A**) the US airport network, (**B**) Ca_Hepth citation network, (**C**) PGP network and (**D**) Power grid network that were discovered by 9 community-finding methods. Each figure for one of the four networks represents the result from one of the nine community-finding methods. For clarity, the x-axes correspond to community sizies, which are ordered from small to large. (**A**) In the US airport network, all of the communities, except four, have variance ratios *ρ* > 0, so that they were marked as leader communities. (**B** and **C**) In the Ca_Hepth citation network and the PGP networks, more leader communities were detected by the nine methods. (**D**) In the Power grid network, more self-organizing than leader communities were identified by eight of the nine algorithms, with Infomap as an exception.

The results from these methods on the four large networks were categorically consistent - these networks contain a large number of communities (Fig. 4), many of which are small. In the analysis, we focused on large communities and ignored small ones with less than $10$ nodes.

The results on these networks showed that the new metric is robust and stable. While different methods might not necessarily generate the same communities, they often produced the same community types (leader or self-organizing) on a given network (Fig. 4). This means that the new metric is robust to tolerate different community detection methods used.

The most interesting result from this analysis of real-world networks is that self-organizing communities tend to be small, and in contrast leader communities are typically large. As shown in Fig. 4, most of the large communities in the four real-world networks (on the right of each panel) have degree variance ratios greater than 1 and were consequently marked as leader communities. This is intuitive as large communities often tend to be difficult to manage so that hierarchical structures with leaders may be more effective for management.

**Figure 5:**
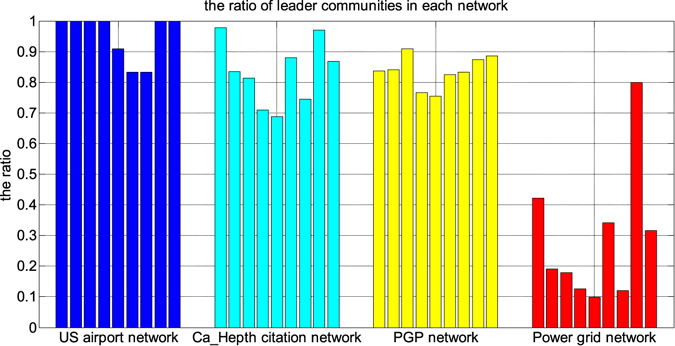
The percentages of leader communities from nine community-finding methods on four real-world networks. Networks are color coded. For each network, nine bars corresponding to nine methods are in the same order as in Fig. 4.

In particular, almost all communities found in the airport network are leader communities (Fig. 4A). All communities discovered by six of the nine methods and the majority of the communities by the remaining three methods are leader communities (Fig. 5). This result is in agreement with the fact that there are large cities or megacities, such as Los Angeles, New York, and Chicago, in different regions of the US, and regional hub airports of major airlines, which correspond to leaders of the communities, have flights connecting to the rest of the airports throughout the regions.

The Ca_Hepth citation network and the PGP network have more leader communities than self-organizing communities (Fig. 4B and C). About $\mathrm{70 \% }$ of the communities of these two networks found by all the methods but one (UVCluster) are leader communities (Fig. 5). Note that the degree variance ratios $\rho$ increase with the community sizes in these two networks. A community in the citation network presumably corresponds to a research field, where there are popular papers that have a large influence on the development of the field. In the PGP network, a community represents a group of people who may know one another, and there are several people in a community who are well connected to the others.

The result from the Power grid network is interesting, in which most communities identified are self-organizing communities (Fig. 4D), meaning that every node plays a similar role in the power grid network. Less than $\mathrm{20 \% }$ of the communities from five of the nine methods (RB, CPM, SCluster, UVCluster and SVI) and no more than half of the communities from three methods (BGLL, OSLOM and COPRA) are leader communities. Informap is an exception on this network. Although most communities from Infomap are leader communities, they do not have large degree variance ratios *ρ* (Fig. 5). In short, most communities of this network are self-organizing communities. Intuitively, a leader community is less stable than a self-organizing community because a failure of the leader(s) may destruct the community or make the community unstable. Such a destructive consequence must be avoided in power grids in order to minimize any catastrophic consequences due to power outage at the leader node(s). Therefore, forming self-organizing communities is a rational design strategy for power grid systems.

## Summary and Discussion

We proposed in this paper a new metric, degree variance ratio *ρ*, that is able to accurately characterize leader communities versus self-organizing communities in complex networks. Two modes of communities evolutions are reflected by these two communities patterns. Nodes in leader communities are more likely to connect to the popular nodes that already have relatively high connectivity, turning them into leaders, while nodes in self-organizing communities do not have a tendency to connect to the popular nodes. We use the words “self-organizing” to refer to the latter case of communities formation and call the resulting communities self-organizing communities. We applied this new metric to benchmark networks with known community structures. The results showed that the new metric outperformed the existing metric and was able to accurately identify leader and self-organizing communities. Furthermore, in combination of nine state-of-the-art community-finding methods, we applied the new metric to four large real-world networks with no community structure information. Although these community-finding methods may not identify the same communities, the new metric provided stable and consistent results on similar communities from different methods, showing that it is robust against perturbations due to different community detecting methods used. Here two communities from different methods are considered similar if they share more than 70% common nodes of the smaller community. Moreover, the results also showed that large communities are more likely to be leader communities and small communities tend to be self-organizing communities. In particular, in the Power grid network, more self-organizing communities are identified than leader communities by our new method. In contrast, nearly all communities in the Airport network have leader nodes, which is in agreement with the reality that many large cities serve as airline hubs with flights connecting to small cities in the regions. The communities in the citation network are mixed, in which most communities are leader communities, which are likely to be relatively mature research fields.

## Materials and Methods

### Networks with known communities

Seven benchmark networks with known community structures were used to test the new and existing metrics for distinguishing leader and self-organizing communities.

#### Lusseau’s bottlenose dolphin network

The Dolphins Social Network, reported by Lusseau, contains 62 dolphins. A link is introduced to connect two dolphins or nodes if the two are observed to be together more often than expected by chance over a period of seven years from 1994 to 2001. The dolphins are mainly divided into the male dolphins and female dolphins, therefore, two communities exist in the network.

#### Zachary’s karate club network

The karate club network^15^ has been used extensively as a benchmark for community detection. A disagreement developed between the coach and the president of the club, which ultimately resulted in the split of the club into two communities.

#### School friendship network

The School Friendship Network was compiled from the National Longitudinal Study of Adolescent Health^21^. It was based on self-reporting from students from grade 7 to grade 12. Grade 9 has two subgraphs of white and black students. The original network can be divided into 6 or 7 communities depending on whether grade 9 is divided into two subgroups or not.

#### College football

The United States college football network, due to Newman^18^, has a node as a team an edge to mean a regular-season game between two teams based on the schedule of 2000 season. The teams are divided into conferences with 8–12 teams each. Games are more frequent between members of the same conference than between members of different conferences.

#### Political blogs

A directed network of hyper links between weblogs on US politics, Polblogs, recorded in 2005 by Adamic and Glance^19^. These blogs can be divided into two groups by learning towards left or right(liberal, conservative) on the political spectrum.

#### Political books

A network of books about US politics published around the time of the 2004 presidential election and sold by the online bookseller Amazon.com ^20^. Edges between books represent frequent co-purchasing of books by the same buyers. The books can be divided into three communities: liberal, conservative and neutral.

#### Word adjacencies

An adjacency network of common adjectives and nouns in the novel David Copperfield by Charles Dickens^22^. The adjectives and nouns constitute two communities of the network.

### Networks with no information of community structures

We considered four large real complex networks, listed in Table 2, on which no information of their communities is available.

### Expected variance of node degrees in the Erdös-Rényi networks

Consider a community *C* of *N* nodes. Let 〈*k*〉 be the average degree of nodes of *C*. We denote $VA{R}_{real}$ and $VA{R}_{rand}$ to be, respectively, the actual and expected variances of node degrees of an ER network with *N* nodes and 〈*k*〉 expected node degree.

We first define the degree variance of community *C*, $VAR(C)$, as follows,

2 $$\begin{array}{ccc}VAR(C) & = & E({{\bf{d}}}_{C}^{2})-E{({{\bf{d}}}_{C})}^{2},\end{array}$$

where ${{\bf{d}}}_{C}$ is the series of node degrees of *C*.

For a small community, we used simulation to approximate the value of $VA{R}_{rand}$. We generated *M* ER networks with the same size *N* and average degree 〈*k*〉, and took the average of their variances as $VA{R}_{rand}$, i.e.,

3 $$VA{R}_{rand}=\frac{1}{M}\sum _{i=1}^{M}VA{R}_{i},$$

where $VA{R}_{i}$ is the degree variance of the ${i}^{th}$ ER network. In the experiments, we considered *M* to be 500 and 1000.

For large networks, we used 〈*k*〉, to be derived below, to approximate $VA{R}_{rand}$. Note that $VA{R}_{rand}$ can be computed as

4 $$\begin{array}{ll}VA{R}_{rand} & =\frac{1}{N}\sum _{i=1}^{N}{({k}_{i}-\langle k\rangle )}^{2}\\  & =\sum _{k=1}^{N-1}P(k){(k-\langle k\rangle )}^{2}\\  & \le \sum _{k=1}^{+\infty }P(k){(k-\langle k\rangle )}^{2}.\end{array}$$

The first step in the equation above comes from the *sum* of ${({k}_{i}-\langle k\rangle )}^{2}$ in different views, the former computes it by adding all of the nodes one by one while the latter computes it via clustering the nodes with the same degree first and then adding all of them. According to Erdös^37^, the distribution of node degrees of a random ER network can be approximated by a Poisson distribution:

5 $$P(k)=\frac{{\lambda }^{k}{e}^{-\lambda }}{k!},$$

where *P*(*k*) is the probability that a randomly chosen node has degree *k*. Combining Equations (5) and (4), we have

6 $$VA{R}_{rand}\le Var(k)=\langle k\rangle \mathrm{.}$$

Equation (6) holds since the variance of a Poisson distribution is *λ*, which is 〈*k*〉. This is reason that the degree variance is smaller than the average degree of ER networks, as shown in Fig. 2. However, for large *k*, *P*(*k*) decrease faster than the increasing of ${(k-\langle k\rangle )}^{2}$, supporting to ignore the items of $k\ge N$ when *N* is large.

Finally, the ratio of degree variance is then defined by

7 $$\rho =\frac{VA{R}_{real}}{VA{R}_{rand}}\approx \frac{VA{R}_{real}}{\langle k\rangle }\mathrm{.}$$

## Electronic supplementary material

Supplementary Information
