# Supplementary material for: Identification of leader and self-organizing communities in complex networks
Source: Sci Rep. 2017 Apr 6;7:704. doi: 10.1038/s41598-017-00718-3 (PMC5429660; doi:10.1038/s41598-017-00718-3)
Supplement: Supplementary file 1 — Supplementary Information [file 41598_2017_718_MOESM1_ESM.pdf]

Identification of leader and self-organising communities in complex networks

Jingcheng Fu[1,3], Weixiong Zhang[2,3], Jianliang Wu[1*]

[1]School of Mathematics, Shandong University, Jinan, 250100, China

[2]College of Math and Computer Science, Institute for Systems Biology, Jianghan University, Wuhan 430056, China

[3]Department of Computer Science and Engineering, Washington University, St. Louis, MO 63130, USA

1 Supplementary Information

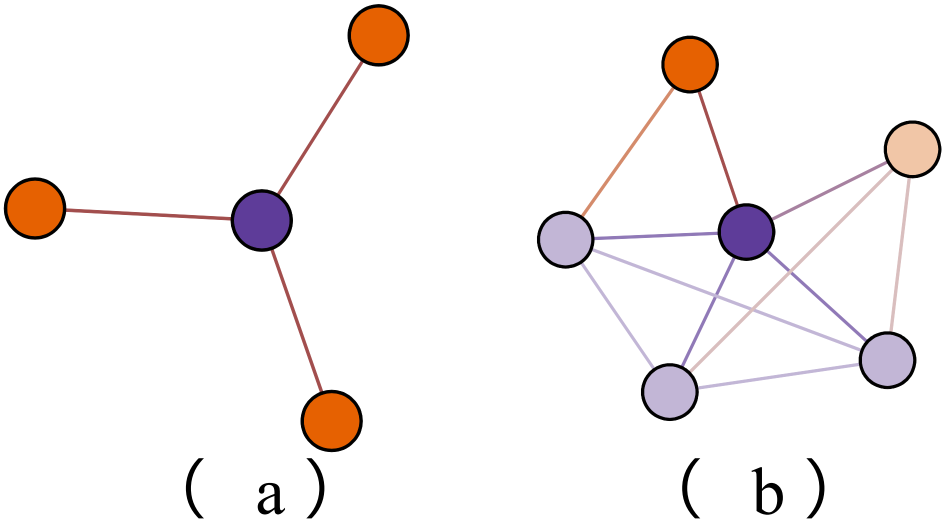

Figure S1: Two communities in School7 network which are detected to be self-organising communities under the old statistic. (a) is also the only one community in School6 which is detected to be a self-organising community under the old statistic. Under our new statistics, these two communities are identified to be leader communities, actually, they are. There is a leader in (a), which is a star. Similarly, the node in the center of (b) can be a leader.

*Corresponding author. *E-mail address:* jlwu@sdu.edu.cn.
